# Core concepts in statistics and research methods. Part 7: regression

**DOI:** 10.1016/j.bjae.2026.02.004

**Published:** 2026-03-20

**Authors:** D. Sidebotham, P.M. Jones

**Affiliations:** 1 Auckland City Hospital, Auckland, New Zealand; 2 University of Auckland, Auckland, New Zealand; 3 Mayo Clinic, Jacksonville, FL, USA

**Keywords:** data interpretation, data reporting, regression, research design, statistics

**Learning objectives**

By reading this article, you should be able to:

• Identify the response and the explanatory variables in a regression model.
• Interpret the regression coefficients (model parameters).
• Identify an appropriate regression model for the clinical question and the data.
• Explain the assumptions of simple linear regression.
• Recognise the differences between linear models and generalised linear models.

**Key points**

• Regression provides a unified framework for statistical inference.
• Regression quantifies the relationship between a response variable and one or more explanatory variables.
• Linear models involve a continuous response and one or more explanatory variables, which may be continuous or discrete.
• Generalised linear models (GLMs) comprise a broad class of regression models that can accommodate virtually all data types.
• Logistic regression is a type of GLM in which the response variable is binary.

The term regression dates back to experiments conducted by British biologist Sir Francis Galton in the 19th century.^1^ Galton was interested in how strongly traits in one generation were inherited in the next. Working with peas, he measured the sizes of mother and daughter peas and plotted the results on a scatterplot. Galton noticed that the line of best fit was approximately straight with a positive slope that was always < 1. Galton interpreted the slope as demonstrating regression to the mean, whereby very large or very small mother peas tended to produce daughter peas of less extreme size. Although regression to the mean is unrelated to Galton’s methodology, the name stuck. Nowadays, we refer to the technique of drawing a straight line of best fit through data points on a scatterplot as simple linear regression.

Since Galton’s work, regression has developed into one of the most important techniques in all of statistics. Regression provides a unified framework for analysing virtually all data. Most standard statistical tests have a regression equivalent (Table 1). Regression can be used with both traditional (frequentist) and Bayesian frameworks. Although Bayesian inference offers distinct advantages, our focus is frequentist statistics, as this is the most widely used approach to statistical inference.

**Table 1:** Data types, standard statistical tests and regression. ANCOVA, analysis of covariance; anova, analysis of variance.

Data type	Standard statistical test	Regression	Example
Continuous response with one continuous explanatory variable	Pearson correlation	Simple linear regression	Relationship between height and weight
Continuous response variable with one binary explanatory variable	*t*-test (independent samples)	Linear regression with categorical explanatory variable (binary)	Effect of drug A *vs* drug B on blood pressure
Continuous response variable with one categorical explanatory variable (three or more levels)	One-way anova	Linear regression with single categorical explanatory variable (multiple levels)	Effect of drugs A, B, and C on blood pressure
Continuous response variable with two or more categorical explanatory variables	Two-way anova	Multivariable linear regression	Effect of diet (categories A, B, and C) and exercise (category low, medium, and high) on weight loss. Specifically, does the effect of diet vary by exercise?
Continuous response variable with one continuous and one categorical explanatory	ancova	Multivariable linear regression	Effect of exercise (categories: low, medium, and high) on weight loss, adjusting for baseline age (continuous)
Continuous response variable with two or more explanatory variables (continuous and categorical)	None commonly used	Multivariable linear regression	Effect of age, sex, diet (categories A, B, and C) and exercise (category low, medium, and high) on weight loss
Binary outcome and a single binary explanatory variable	Fisher’s exact test; *z*-test for two proportions; χ^2^ test	Logistic regression	Effect of prone positioning on mortality
Binary outcome with two or more explanatory variables (continuous and categorical)	None commonly used	Multivariable logistic regression	Effect of age, sex, ethnicity and weight on mortality
Count response with multiple explanatory variables	None commonly used	Poisson regression; negative binomial regression	Number of episodes of vomiting with three different antiemetics
Categorical response (non-ordered) with multiple explanatory variables	None commonly used	Multinomial regression	Choice of treatment (medical *vs* surgery *vs* none) based on age and disease severity

This article is part of a series in *BJA Education* on statistics and study design.^2^, ^3^, ^4^, ^5^ Concepts from earlier articles that will assist in understanding the material presented here include the difference between variables and parameters, variance, standard error, test statistics, null distributions, hypothesis tests, *p*-values and confidence intervals (CIs).

We introduce regression by way of simple linear regression and progress through to generalised linear models (GLMs), an important class of regression models that includes logistic regression.

## What is regression?

Regression is a statistical methodology that quantifies how one variable (the *response variable*) changes in relation to another variable (or variables), called an *explanatory variable*. For instance, we might be interested in how weight varies with height or how the rate of postoperative respiratory complications varies with age and the use of sugammadex. The words explanatory and response simply indicate the direction of a potential effect, and do not imply causality. Several other terms are in common use (Table 2).

**Table 2:** Alternative terms for the explanatory and response variables. In some circumstances, the term covariate indicates any explanatory variable; in other circumstances, covariate refers exclusively to a continuous explanatory variable and factor is used to refer to a categorical explanatory variable. In this article, we limit the term covariate to a continuous explanatory variable.

Response variable	Explanatory variable	Use
Dependent	Independent covariate (factor)	Experimental science
Outcome	Predictor	Epidemiology
	Exposure	
	Risk factor	
Target	Feature	Machine learning
Label	Input	
Regressand	Regressor	Mathematics and statistics

## Simple linear regression

**Fig 1:**
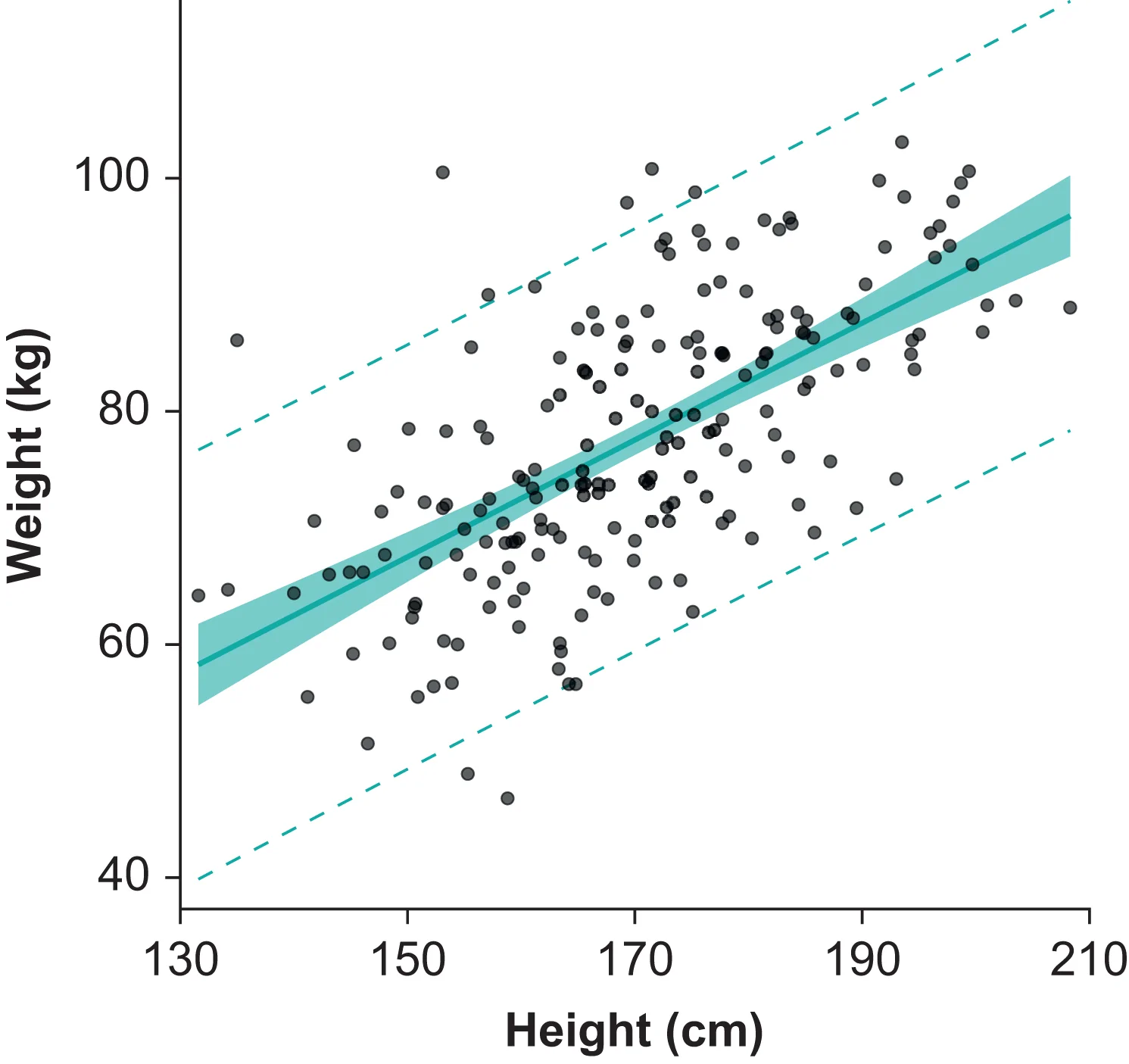
**Scatterplot of heights and weights.** The data are from a simulated dataset of 200 adult heights, weights and sex. Height is the explanatory variable, shown on the x-axis, and weight is the response variable, shown on the y-axis. The green solid line shows the shows the fitted regression line (y=−7.8+0.5x). The green shading shows the 95% CI around the regression line. The green dashed lines show the lower and upper bounds on the 95% prediction interval. The 95% CI around the regression line shows the plausible range for the *expected* (i.e. mean) weights for given heights. The 95% prediction interval shows the plausible range of weights of an *individual* with a given height.

Simple linear regression quantifies the relationship between one continuous explanatory variable (e.g. height) and one continuous response variable (e.g. weight). A continuous explanatory variable is called a covariate. When considering the relationship between height and weight, it is plausible that height affects weight but not the reverse. So, height would be the explanatory variable and weight the response variable. On a scatterplot, values for the explanatory variable are along the *x*-axis and values for the response variable along the *y*-axis (Fig. 1). A commonly used shorthand is to write the model as y∼x*,* where *y* is the response and *x* is a covariate. The observed data points ($x_{i},y_{i}$) are used to calculate a line of best fit using a technique called *ordinary least squares*. Ordinary least squares minimises the sum of the squared vertical distances between the observed data points and the fitted regression line (i.e. the residuals, described below) to provide a straight line that best fits the data. Squaring is used to account for negative (below the line) and positive (above the line) observed data points. The line of best fit is the *fitted model* and has the following form:

(1) $$y={\hat{\beta }}_{0}+{\hat{\beta }}_{1}x$$

In equation (1), *y* is the expected (mean) value for the response when the explanatory variable takes the value *x*. The terms ${\hat{\beta }}_{0}$ and ${\hat{\beta }}_{1}$ are the fitted regression coefficients. The hat notation indicates they are sample estimates of the true population coefficients (β_0_, β_1_). The regression coefficients are the *parameters* of the model. Collectively, the term to the right of the equals sign is called the *linear predictor*.

We will illustrate simple linear regression using a simulated dataset of height, weight and sex for 200 adults. Figure 1 shows a scatterplot of the height and weight data. We used the data to develop the model weight∼height in the statistical programming language R.^6^ The fitted regression line is y=−7.8+0.5x.

### Interpreting the regression coefficients

The coefficient ${\hat{\beta }}_{1}$ is the estimate of the *slope parameter* and represents the expected change in the response for a single step change in the explanatory variable. So, for the model weight∼height, the expected increase in weight for every 1 cm in height is 0.5 kg. The coefficient ${\hat{\beta }}_{0}$ is the estimate of the *intercept parameter* and represents the expected value for the response when the explanatory variable is zero. For the model weight∼height, the expected weight when height is 0 cm is –7.8 kg. For many regression models, the intercept falls well outside the range of sample data and has no meaningful interpretation.

### Assumptions

**Fig 2:**
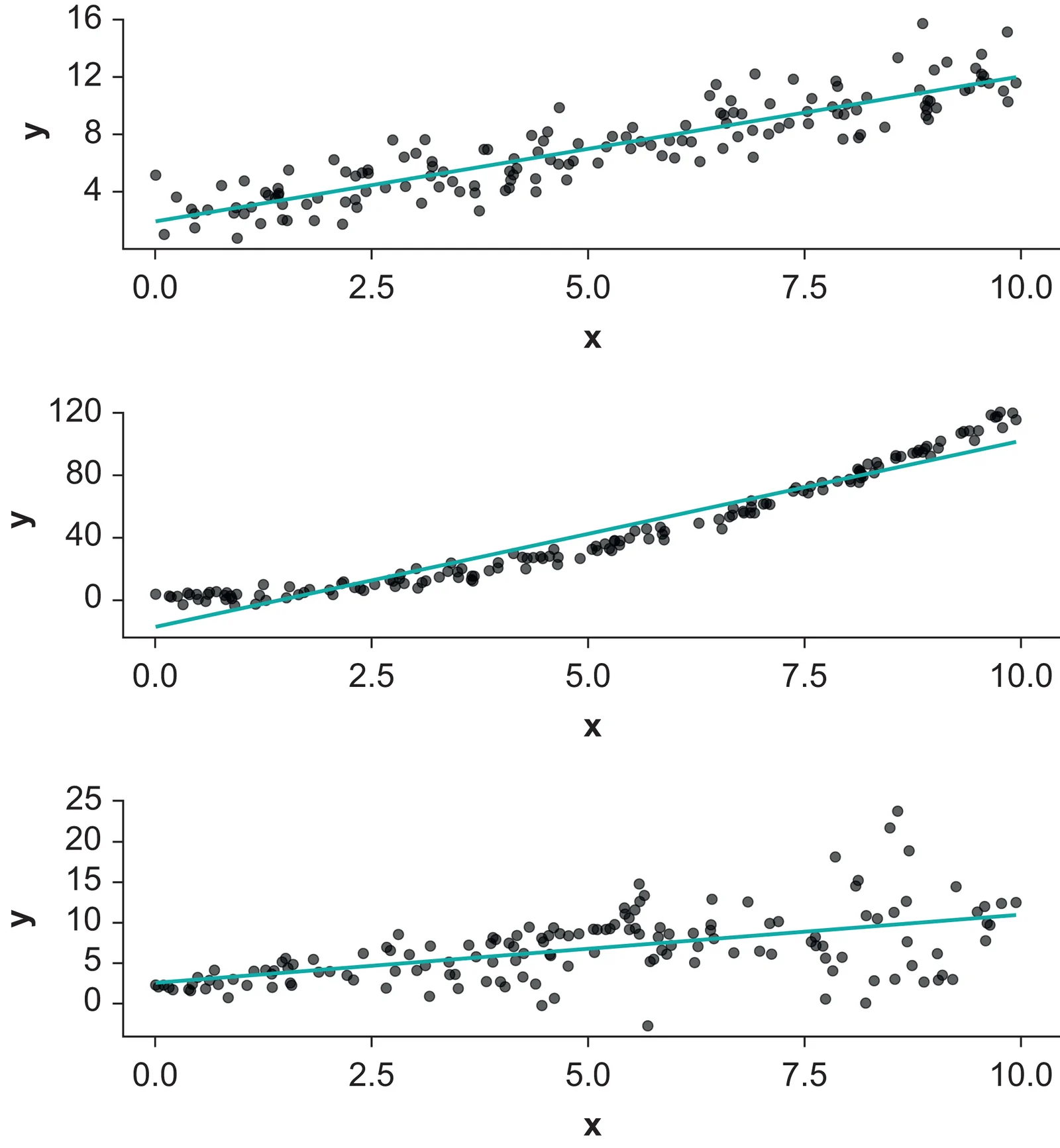
**Simulated scatterplots of an explanatory variable (x-axis) and a response variable (y-axis).** Top panel. The relationship between the two variables is plausibly linear and the scatter around the regression line is plausibly constant, indicating constant variance for the residuals; equation (2) in the text. Middle panel. The relationship between the variables appears to be non-linear, suggesting that a linear model is not appropriate. Transforming one or both of the variables may satisfy the assumption of linearity. Bottom panel. The relationship between the variables is plausibly linear, but the scatter around the regression line increases with increasing x-values. The increased scatter at high x-values suggests the variance of the residuals is not constant, violating one of the assumptions of linear regression.

There are three key assumptions for linear regression. First, each observation is independent; that is to say, one observation does not affect another. Second, the relationship between the explanatory and response variables is linear. Figure 2 shows scatterplots for some hypothetical data. In the top and bottom panels the relationship is plausibly linear, whereas in the middle panel the relationship is non-linear. Third, the residuals are normally distributed with zero mean and constant variance. A *residual* ($r_{i}$) is the vertical difference between an observed response value ($y_{i}$) and the expected value ($\hat{y_{i}}$):

(2) $$r_{i}=y_{i}-\hat{y_{i}}$$

In Figure 2 (bottom panel) the spread of observed y values gets larger as *x* increases, suggesting that the variance of the residuals is not constant. The assumption that the residuals are normally distributed with zero mean and constant variance can be assessed using a residuals plot or a normal probability plot.

In many datasets, the assumptions of linearity and constant variance are not satisfied. However, all is not lost, as *transforming* one or both of the variables may satisfy the assumptions. For right-skewed data, which are common in medicine,^2^ one option is to take the natural logarithm (i.e. to base *e*) of the response variable, which may satisfy the assumptions of linearity and constant variance. If the assumptions are not satisfied, inferences drawn from the model may not be valid.

### Predicting values for the response variable

Regression models are used for making predictions and for testing for associations. In Figure 1, the fitted regression line passes through the point (170, 77.2), meaning that when height is 170 cm, the expected weight is 77.2 kg. When making predictions, we must be mindful of the sources of variability in the model. One source of variability is the uncertainty over the position of the true (population) regression line. The fitted regression coefficients are sample estimates of population values and consequently subject to sampling variability. A CI around the regression line provides a plausible range for the *expected y* value for a given *x* value (Fig. 1). However, when predicting an *individual y* value for a given *x* value, we must account for the scatter around the regression line (i.e. the residuals). A *prediction interval* provides a plausible range for the actual *y* value for a given *x* value (Fig. 1).

*Interpolation* is the process of making predictions on the response variable when the explanatory variable lies within the range of the sample data. When the explanatory variable lies outside the range of the sample data, making predictions on the response is called *extrapolation*. Interpolated predictions are more robust than extrapolated predictions.

### Testing for a relationship

In medical research, identifying associations is of far greater interest than making predictions. We start with the null hypothesis (H_0_) that *x* does not affect *y*. If H_0_ is true, we would expect a random scatter of data points with a horizontal fitted regression line (i.e. β_1_ = 0). The test statistic (*t*) has the usual form of the sample estimate divided by its standard error (se)^2^:

(3) $$t=\frac{{\hat{\beta }}_{1}}{SE}$$

For linear models, the null distribution is a *t* distribution. (The null distribution and *t* distributions were explained in Parts 1 and 4, respectively.^2^^,^^4^) Using standard formulae, a 95% CI can be calculated around the estimate (i.e. around ${\hat{\beta }}_{1}$), which gives a plausible range for the population value (i.e. for β_1_). If *p* ≤ 0.05 or the 95% CI excludes the null value (i.e. 0), we reject H_0_ and conclude that there is a relationship between the explanatory and response variables at the 5% significance level. For the model weight∼height, the *p*-value is very small (< 0.001), indicating the data provide very strong evidence that height is associated with weight.

## Linear regression with a single categorical explanatory variable

**Fig 3:**
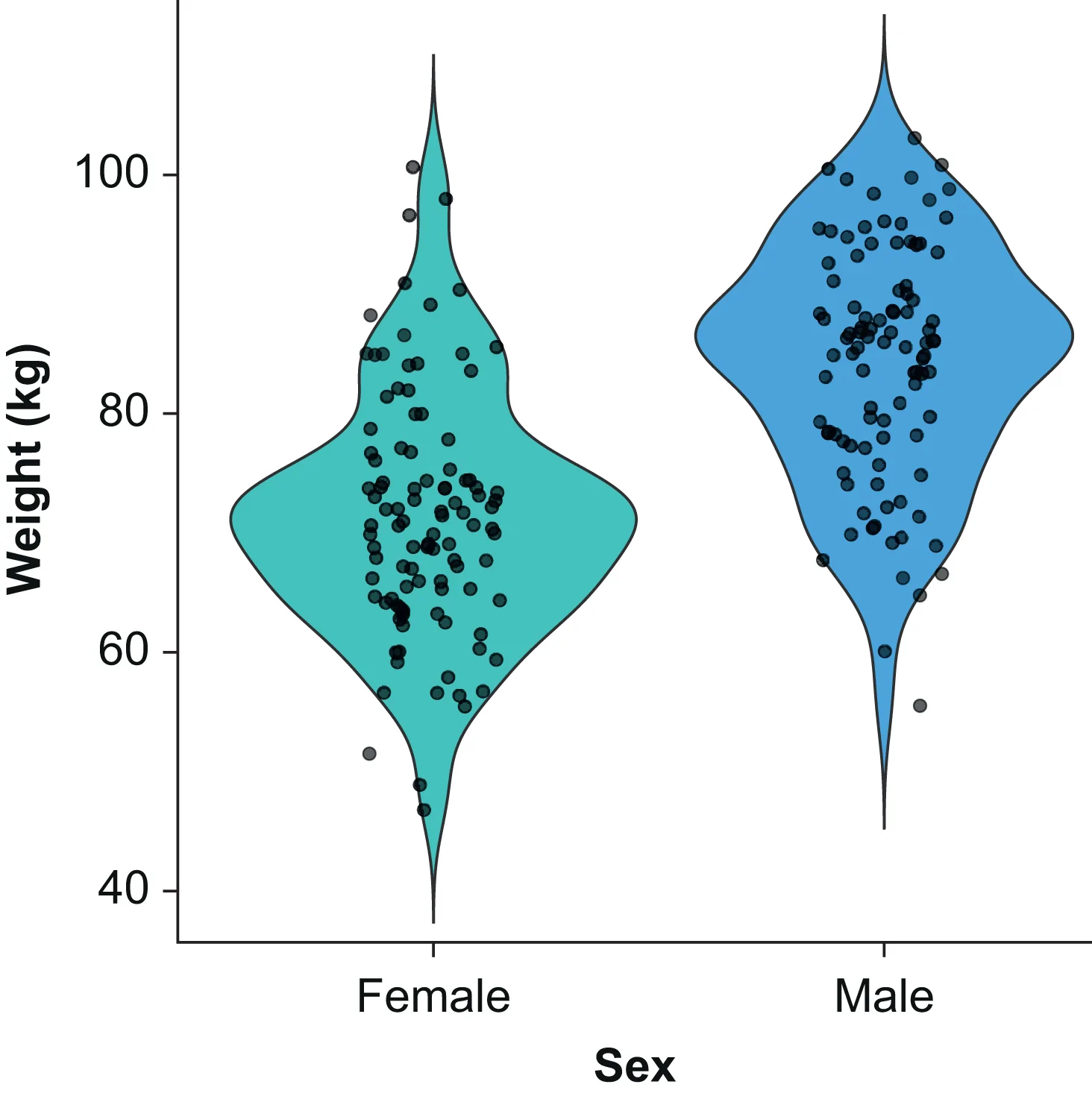
**Comparative violin plots.** The data are from the same simulated dataset shown in Figure 1. The figure shows a violin plot, which displays the distribution of data as a smoothed, mirrored density curve—wider sections indicate where more data points are concentrated. By comparison, a boxplot (not shown) is a vertical rectangle spanning from the lower quartile (25th percentile) to the upper quartile (75th percentile), with a horizontal line at the median and 'whiskers' extending to show the tails of the distribution. Boxplots summarise data with key statistics, whereas violin plots reveal the full shape of the distribution, including multiple peaks or asymmetry. The left plot shows the distribution of weights by female sex and the right plot shows the distribution of weights by male sex. Overall, females tend to weigh less than males.

So far we have only considered simple linear regression involving a continuous response and a continuous explanatory variable (covariate). However, we may be interested in a categorical explanatory variable. Categorical explanatory variables are called factors (e.g. sex) and the values the factor can take are called levels (e.g. male and female). The relationship between a continuous response variable and a factor can be seen using comparative box or violin plots (Fig. 3). Factors are typically represented by upper case letters. So we can denote the model as y∼A.

For our simulated dataset, we might be interested whether sex influences weight. The model is denoted weight∼sex. We defined female sex as level 0 and male as level 1. As the ordering of the levels is arbitrary (i.e. the categories are nominal^2^), we could have defined male as level 0. The regression coefficient tells us the effect a particular level has on the response variable *relative to the reference level* (i.e. level 0, female sex in this example).

For weight∼sex, the fitted model is y=71.4+12.7z*,* where *z* is a variable that takes the value 0 when sex is female and 1 when sex is male. Thus, the expected weight for females (*z* = 0) is 71.4 kg and for males (*z* = 1) is 71.4+12.7=84.1 kg. The coefficient for the effect of male weight (12.7) is called an effect coefficient, and in this case indicates that males have an expected weight 12.7 kg heavier than females.

### Testing for a relationship

As with simple linear regression, we can test the null hypothesis that the effect coefficient equals 0. For our model weight∼sex*,* the *p*-value is very small (< 0.001), indicating the data provide very strong evidence that sex is associated with weight.

## Multivariable linear regression

Multivariable linear regression extends simple linear regression to include multiple explanatory variables, which may be covariates, factors or both. For example, the model $y\;∼\;x_{1}+x_{2}+A+B$ involves two covariates and two factors. As with simple linear regression, the response variable is continuous.

Before going further, it is worth commenting on terminology. When there is one response variable and multiple explanatory variables, the term multivariable (or *multiple) regression* is appropriate.^7^ The term *multivariate regression* refers to situations when the response variable is measured multiple times, often involving longitudinal data on the same group of patients. When a model has one explanatory variable it is common to use the term univariate, but for consistency we will use the term *univariable*.

Consider our simulated dataset of weight, height and sex. It is plausible that both height and sex independently affect weight. Alternatively, it is possible that the effect of sex arises because, on average, women are not as tall as men; that is to say, weight is a confounder for the relationship between sex and height. We can investigate the question using the model weight∼height+sex. The fitted model from our simulated dataset is y=0.04+0.43x+9.8z. Again, *x* represents height and *z* is a variable that takes the value 0 when sex is female and 1 when sex is male.

### Visualising multivariable models

**Fig 4:**
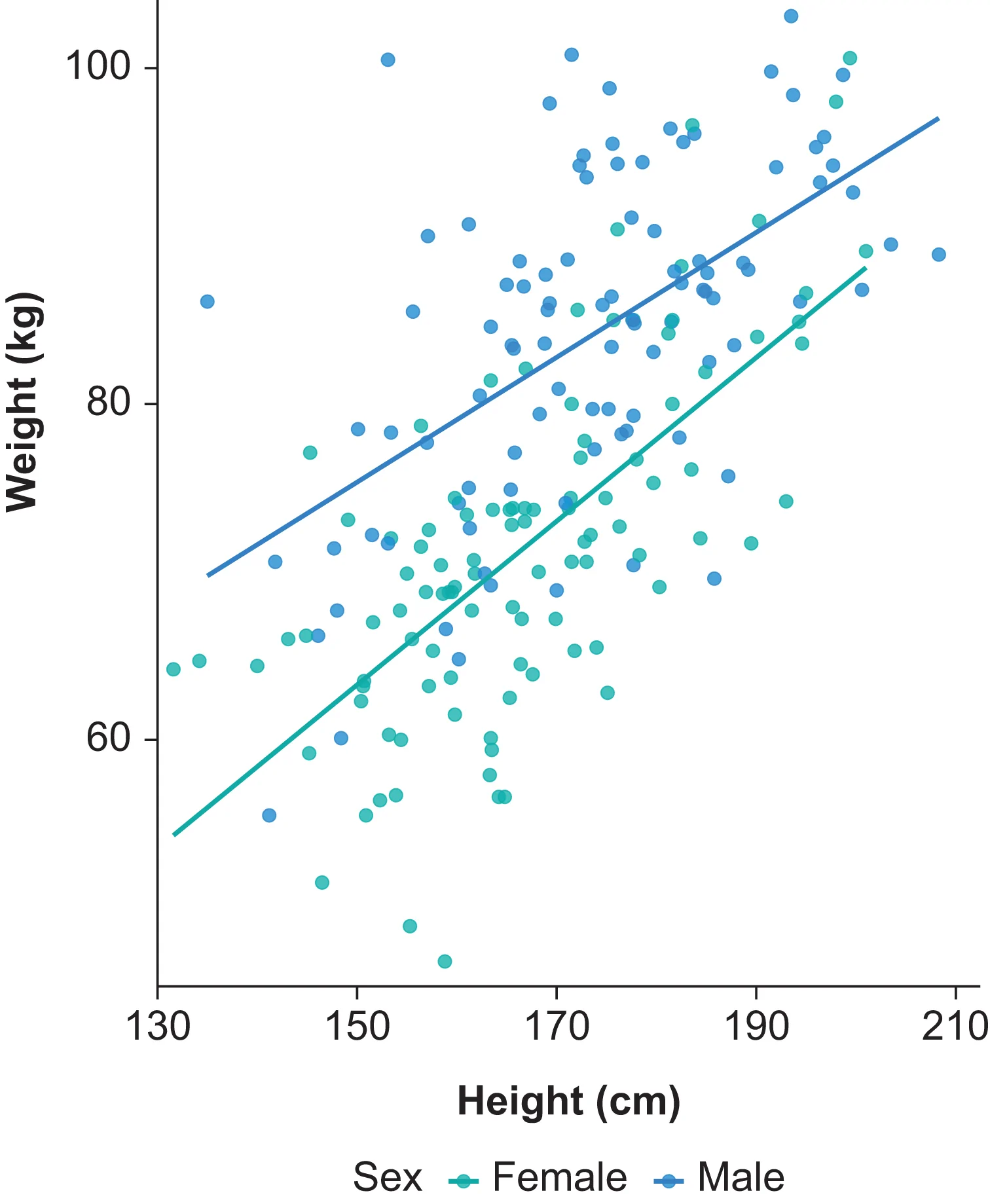
**Scatterplot of heights and weights colour-coded by sex.** The scatterplot shows the same data as Figure 1, except now female sex is indicated by green dots and male sex by blue dots. Separate regression lines are shown for females (green) and males (blue). Notice that the slopes of the two regression lines are different, suggesting that the effect of sex on weight is different for males and females. Non-parallel slopes may indicate that the model fit may be improved by adding an interaction term between sex and height (see text for details).

Figure 4 shows a scatterplot for the model weight∼height+sex, where females and males are coded green and blue, respectively. For models involving multiple covariates and factors, it is not possible to represent the entire model on a single plot, and multiple scatterplots and violin (or box) plots are required to show the effects of individual explanatory variables.

### Interpreting the coefficients in a multivariable regression model

For multivariable regression, we interpret the regression coefficients as the effect a particular explanatory variable has on the response when all the other explanatory variables are held at a constant value.

For the model weight∼height+sex*,* the effect coefficient for male sex is 9.8, meaning we expect males to be, on average, 9.8 kg heavier than females of the same height. Note that the effect of male sex (9.8 kg) is less than that for the model weight∼sex *(12.*7 kg*)* because some of the effect of male sex arises because males are, on average, taller than females.

For the model weight∼height+sex*,* the expected weight for a male (*z* = 1) who is 174 cm tall is 0.04+0.43×174+9.8=84.7 kg, and that for a female (*z* = 0) is 0.04+0.43×174=74.9 kg.

As with univariable regression, we can test for associations by calculating *p*-values and CIs for the regression coefficients (see below).

### Interactions

In Figure 4 the regression lines for males and females are not parallel, which suggests that the effect of height on weight is different for males and females. In this circumstance, we say height and sex *interact*. If the interaction is important, predictions can be improved by including an interaction term in the model (weight∼height+sex+height∗sex*)*. If the *p*-value for the interaction term is small or the overall model fit (see below) is significantly improved, the interaction term should be included. The *p*-value for the interaction height∗sex was 0.12 (i.e. not small) and the model fit was similar with and without the interaction term. The principle of parsimony states that if two models explain the data roughly equally well, we should choose the simpler model. So, we should use the model weight∼height+sex *in* preference to weight∼height+sex+height∗sex.

### Independent predictors and a note of caution

For the model weight∼height+sex, the *p*-values associated with the regression coefficients for both height and male sex (relative to female sex) are very small (< 0.001). It is common to interpret these findings as indicating that height and sex are both independent predictors of weight. However, we should be cautious when claiming an explanatory variable is an independent predictor of the response. First, declarations of statistical significance do not reliably identify real effects, especially when the *p*-value is close to 0.05.^8^^,^^9^ Second, adjusting for confounding in observational datasets is very difficult.^3^ For observational datasets containing multiple variables, an almost infinite number of models are possible, and different models yield different results.^10^ Hidden confounder variables (Donald Rumsfeld’s ‘unknown unknowns’^11^) may not have been included in the model, giving the false impression that a particular explanatory variable independently changes with the response. Third, multivariable regression models generate multiple *p*-values, which increases the chance of a false positive result. For instance, a model that generates 20 *p*-values that is applied to a dataset where there are no real effects has a 64% chance of identifying at least one false positive result.^12^

## Generalised linear models

So far we have considered models involving a continuous response variable, termed *linear models*. However, we are often interested in other types of responses; for instance, a binary outcome (e.g. dead *vs* alive), a count (e.g. the number of episodes of vomiting) or a score on an ordinal scale (e.g. a Likert scale). *Generalised linear models* are a class of regression models that can accommodate virtually all types of response variable.

A key feature of GLMs is the *link function*. For simple linear regression, the response variable and the linear predictor can (theoretically) take any value. However, if the response variable is constrained to take only positive values (e.g. a count) or values between 0 and 1 (e.g. a probability), we require a link function that transforms the linear predictor to match the scale of the response.

Because of its importance in medical research, we will focus on one GLM, logistic regression.

## Logistic regression

Logistic regression is used when the response variable is binary (e.g. dead *vs* alive). We will illustrate the principles of logistic regression using a simulated dataset of patients undergoing venoarterial extracorporeal membrane oxygenation (VA-ECMO) after cardiac surgery (Box 1). The dataset contains information on age, sex, indication for VA-ECMO, chronic kidney disease (CKD), and mortality.

Box 1 **Logistic regression.**

We simulated a dataset of 400 patients receiving VA-ECMO after cardiac surgery. The response was mortality. Overall mortality was 273/400 (68%). The explanatory variables were age, indication for VA-ECMO, sex and the presence of CKD. We structured the dataset so that mortality was higher in older patients, those undergoing non-transplant cardiac surgery, and in patients with CKD. We made CKD more common in older patients.

**Explanatory variables**

Age (covariate), median (interquartile range), 42 (28–56) yrs

Indication (factor)

• Level 0: transplant (91/154 [59%] died)
• Level 1: non-transplant (182/246 [74%] died)

Sex (factor)

• Level 0: female (101/148 [68%] died)
• Level 1: male (172/252 [68%] died)

CKD (factor)

• Level 0: no (134/218 [61%] died)
• Level 1: yes (140/182 [76%] died)

**Models**

mortality∼age

mortality∼sex

mortality∼indication

mortality∼CKD

mortality∼age+sex+indication+CKD

**Table:** 

Results				
Variable	Univariable models		Multivariable model	
	Unadjusted OR (95% CI)	*p*-value	Adjusted OR (95% CI)	*p*-value
**Age** (per year)	1.05 (1.03–1.06)	< 0.001	1.04 (1.03–1.06)	< 0.001
**Sex** (male relative to female)	1.00 (0.64–1.54)	0.99	1.03 (0.64–1.63)	0.95
**Indication** (non-transplant relative to transplant)	1.97 (1.28–3.03)	0.002	2.26 (1.43–3.59)	< 0.001
**CKD** (yes relative to no)	2.03 (1.31–3.16)	0.002	1.42 (0.88–2.30)	0.15

**Comment**

With univariable modelling, increased age, non-transplant surgery and CKD were statistically significantly associated with increased mortality at the 5% significance level. With multivariable modelling, only increased age and non-transplant surgery were statistically significant. According to the model, increased age and non-transplant surgery are independent predictors of mortality.

Alt-text: Box 1

To investigate the effect of age on mortality, we assign age as the explanatory variable and mortality as the response, denoting the model mortality∼age. We are modelling the probability (*p*) of death (i.e. the chance of dying). The linear predictor for the fitted model has the same form as equation (1) (i.e. ${\hat{\beta }}_{0}+{\hat{\beta }}_{1}x$), where *x* is the patient’s age in years. We require a link function that relates the linear predictor (which can take any value) to the response (a probability, constrained to take values between 0 and 1). The logit function is the standard link function for logistic regression. The logit function has the form $\ln\left(\frac{p}{1-p}\right)$, where ln refers to the natural logarithm. For the model mortality∼age*,* the fitted regression equation is logit(p)=−1.1+0.05x. Thus, for a 60-yr-old patient, the expected probability of dying with VA-ECMO involves solving logit(p)=−1.1+0.05×60 for *p*, which is approximately 0.65 (65%).

### Interpreting the regression coefficients

Using the logit function has a very useful consequence when interpreting the regression coefficients. When the explanatory variable is continuous (e.g. the model mortality∼age*),* the intercept coefficient (β_0_) represents the natural logarithm of odds of the outcome when the explanatory variable is zero (which often has no clinical meaning) and the slope coefficient (β_1_) represents the change in the natural logarithm of the odds of the outcome for a one-unit increase in the explanatory variable. When the explanatory variable is binary (e.g. the model mortality∼sex*)*, the intercept represents the natural logarithm of the odds of the outcome in the reference group and the slope represents the difference in the natural logarithm of the odds between the groups.

Exponentiating the coefficients (i.e. *e* raising to the power of the coefficient) gives the odds of the event or the odds ratio (OR) for the outcome. For instance, for the model mortality∼age, the intercept is –1.1, indicating that estimated odds of death when age is 0 is *e*^–1.1^ = 0.33 (which has no clinical meaning) and the intercept is 0.05, indicating the estimated OR for the effect of a 1-yr increase in age is *e*^0.05^ = 1.05 (Box 1). For a randomised trial involving two groups and a binary outcome, the exponentiated value for the intercept represents the estimated odds of the event rate in the reference (control group) and the exponentiated value for the slope coefficient represents the estimated OR for the treatment effect. (Odds and ORs were discussed in Part 1.^2^) A CI around the estimated OR can be constructed using standard methods.^2^

### Multivariable logistic regression

The same principles of univariable logistic regression can be used when there are multiple explanatory variables. Returning to our simulated dataset of patients undergoing VA-ECMO, we can see from Box 1 that with univariable logistic regression, age, non-transplant surgery and the presence of CKD are statistically significantly associated with mortality at the 5% significance level. We structured the dataset such that CKD was more common in older patients. Thus, age is a confounder for CKD. In the multivariable model (mortality∼age+sex+indication+CKD)*,* age and non-transplant surgery remain statistically significant, whereas CKD is no longer significant. Thus, the multivariable model suggests that in this hypothetical group of patients the presence of CKD is not an independent predictor or mortality following VA-ECMO after cardiac surgery.

### Applications of logistic regression

Univariable logistic regression is a common way of analysing data from randomised trials. For instance, Heddle and colleagues^13^ used logistic regression to analyse the outcome of short- or long-term blood storage on mortality. In their model, the explanatory variable was a two-level factor (short *vs* long storage) and the response was in-hospital mortality.

Conversely, multivariable logistic regression is commonly used when analysing observational datasets to identify whether a variable of interest is an independent predictor of a binary outcome. For instance, Suleiman and colleagues^14^ used multivariable logistic regression to determine whether the choice of neostigmine or sugammadex was predictive of postoperative respiratory complications. To adjust for confounders, the authors included many other covariates (e.g. age, BMI) and factors (e.g. sex, hospital, ASA score, surgery type) in the models. The adjusted OR (95% CI) was 1.01 (0.94–1.08). As the 95% CI includes the null value (an OR of 1), the result does not support the hypothesis that the choice of reversal agent is an independent predictor of postoperative respiratory complications (in their population at the 5% significance level).

## Model fit

Model fit refers to how well the fitted model explains the data. Indices of model fit depend on the type of regression model. For linear models, model fit is quantified by the *R*^2^ and *F* statistics. *R*^2^ is the proportion of the variance in the response that is explained by the explanatory variable(s), with possible values ranging between 0 and 1. The adjusted *R*^2^ is a modification that accounts for model complexity, penalising models that contain many explanatory variables. When comparing two models, the one with the higher adjusted *R*^2^ explains the data best. The *F*-statistic is another measure of model fit. In general, the larger the *F*-statistic (and, therefore, the smaller the *p*-value), the better the model fits the data. If a simple model (e.g. weight∼height) is nested within a more complex model (e.g. weight∼height+sex) we can compare the *F* statistics of the two models using an analysis of variance (anova) test. If the *p*-value is small, the more complex model explains the data better than the simpler model.

For logistic regression, a common measure of model fit is the Akaike information criterion (AIC). As with the adjusted *R*^2^, the AIC penalises unnecessarily complex models. When comparing two models, the one with the *lower* AIC explains the data best.

## Variable selection

Selecting explanatory variables is a key consideration when building multivariable models. There are no fixed rules and the topic is subject of debate.^15^ We should be guided by the principles of parsimony and biological plausibility (common sense). As a rule of thumb, there should be a minimum of 10–15 occurrences of the outcome (e.g. deaths) per explanatory variable.^15^^,^^16^

A common approach is to only include explanatory variables that are statistically significant with univariable modelling; however, this risks omitting explanatory variables that are confounders but are not themselves statistically significant.^17^ Another approach is to compare indices of model fit. For simple datasets, where there are only a limited number of plausible models, we can use anova to compare nested models. For high-dimensional datasets (i.e. datasets with many variables), it is common to use stepwise regression, which is an algorithmic process where variables are progressively added or removed to obtain an optimal model based on an index of model fit (e.g. the AIC). Readers should interpret these models with caution as they are data-driven, not hypothesis-driven.

*Multicollinearity* is an important consideration when selecting explanatory variables. Multicollinearity is when two or more explanatory variables are highly correlated. For instance, in a dataset involving patients in the ICU, measures of illness severity (e.g. the Acute Physiology and Chronic Health Evaluation [APACHE] score and the Sequential Organ Failure Assessment [SOFA] score) are likely to be highly correlated. If both variables are included, the model will underestimate the effects of each variable (APACHE, SOFA) even though illness severity might affect the outcome. Multicollinearity can be investigated by examining a correlation matrix, which is a plot showing the correlation coefficients between all the variables. If two explanatory variables are highly correlated, only one should be included in the model.

### Underfitting and overfitting

A multivariable model that poorly explains the data is *underfitted*. Underfitted models have low predictive power and may not identify confounding. Increasing the sample size and including more explanatory variables will likely improve the model fit. A model that perfectly explains the data is also problematic, as *overfitted* models incorporate random noise that is unique to the sample. Overfitted models are not generalisable to the population and have poor predictive power. Increasing the sample size and reducing the number of explanatory variables prevents overfitting.

## Mixed effects models

The models discussed above assume observations are independent and identically distributed. However, in multicentre trials, outcomes amongst patients from the same centre may be correlated owing to shared characteristics. Specifically, the control group event rate (β_0_) and relationship between the intervention and the outcome (β_1_) may differ across centres. Failure to account for differences across treatment centres may lead to an underestimate of the standard error, which in turn leads to a falsely narrow CI.

One approach to deal with clusters is to use a mixed effects model.^18^ A mixed effects model allows the regression coefficients (β_0_, β_1_) to vary across different levels of a factor (e.g. treatment centre). The overall relationship between the explanatory variable and the response is called a *fixed effect*. The values for the regression coefficients across different levels of a factor are called *random effects*.

Although there is no fixed rule, at least five levels of a factor are required to reliably estimate the group-level variation.^19^ Thus, for a two-level factor (e.g. sex) an interaction term would be appropriate, not a mixed effects model.

**Fig 5:**
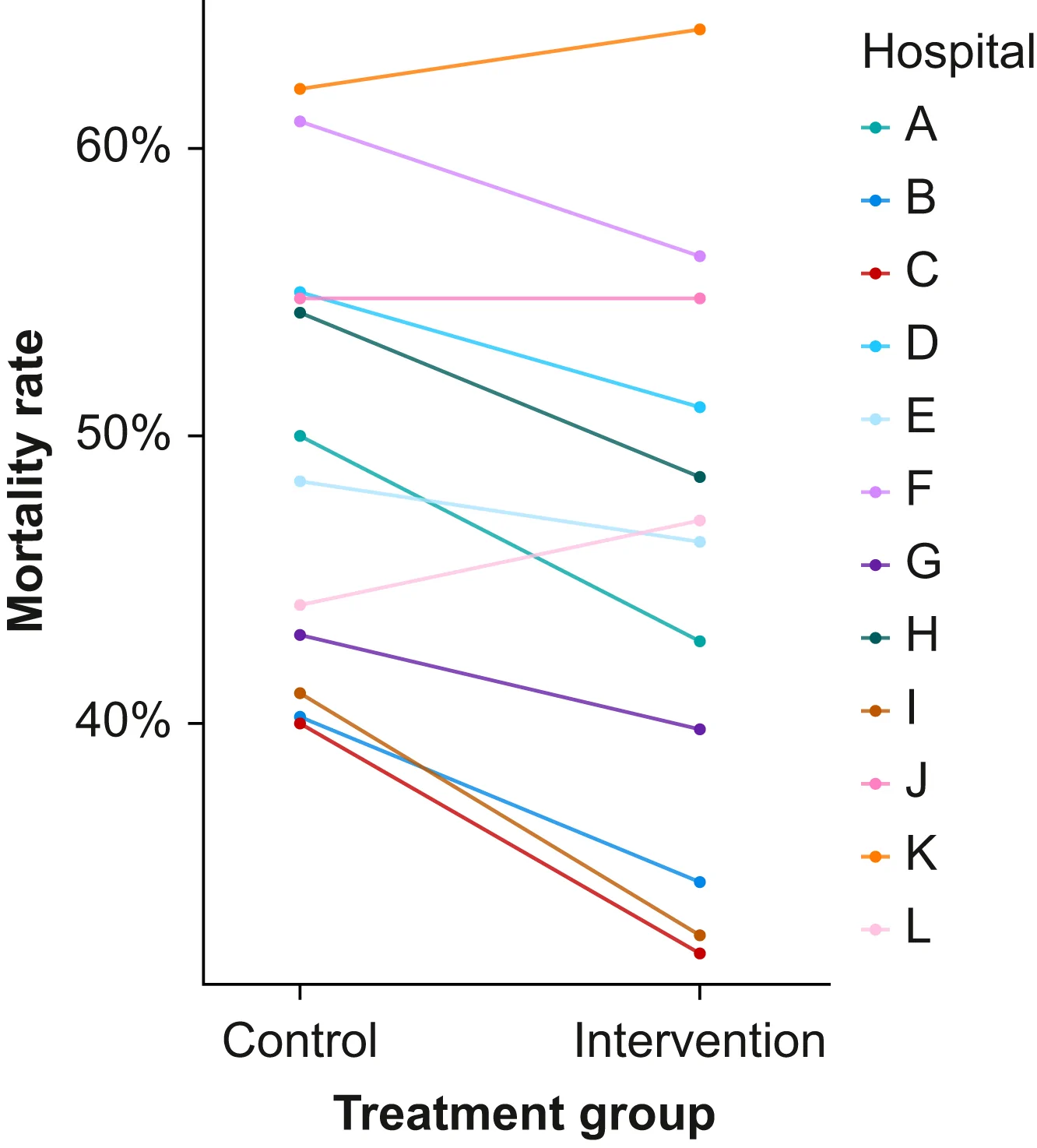
**Slope plot showing the effect of treatment centre on the baseline mortality and treatment effect.** The data are from a simulated multicentre randomised trial (3065 patients, 12 treatment centres). Patients were randomly allocated to a control group or an intervention group. The outcome was a binary event (mortality). The control group mortality is shown on the left and the intervention group mortality is shown for each of 12 treatment centres. See Box 2 for details.

To demonstrate mixed effects models, we created a simulated dataset of 3065 patients at 12 treatment centres who were randomly allocated to an intervention or a control group. The response variable was a binary event (mortality). We designed the dataset so that the baseline event rate and treatment effect varied across treatment centres (Fig. 5). Box 2 compares a standard logistic regression model (mortality∼group*)* with a mixed effects model (mortality∼group *+ group|centre).* The notation *group|centre* means the intercept (β_0_) and slope (β_1_) coefficients are allowed to vary by treatment centre. In Box 2 the estimated treatment effect is almost identical for the two models, but the CI is wider for the mixed effects model because the mixed effects model accounts for the additional variability arising from differences across treatment centres.

Box 2 **Mixed effects model vs standard logistic regression model.**

We simulated a dataset for a multicentre randomised trial involving 3065 patients at 12 hospitals. Patients were randomly allocated to an intervention or a control group and the outcome was mortality. The overall mortality rates for the control and intervention groups were 49.1% and 45.2%, respectively (risk difference –3.9%). We chose the effect size so the result would be close to the usual significance threshold of *p* = 0.05.

We designed the dataset so that the control and intervention group mortality varied across treatment centres by 40.0–62.1% and 32.0–64.1%, respectively (Fig. 5).

**Models**

We ran two logistic regression models:

1. Standard logistic regression: mortality∼group
2. Mixed effects model: mortality∼group *+ group|centre*

**Table:** 

Results				
Model	Standard error	OR (95% CI)	Interval width	*p*-value
Standard logistic regression	0.07	0.86 (0.74–0.99)	0.33	0.03
Mixed effects logistic regression	0.08	0.86 (0.73–1.00)	0.37	0.05

**Interpretation**

Notice that the estimate (OR = 0.86) is less than 1, indicating a reduced odds of death in the intervention group and is the same for the two models. The standard error is larger for the mixed effects model, as it incorporates the additional variation owing to the treatment centre effect. The 95% CI is wider and the p-value slightly larger for the mixed effects model.

Alt-text: Box 2

Mixed effects models are commonly used for analysing data from multicentre randomised trials. In the trial comparing the effect of duration of blood storage on mortality, described earlier, Heddle and colleagues^13^ used a mixed effects logistic regression model with differences across study centre and blood type treated as random effects.

## Alternatives to multivariable regression

An alternative to multivariable regression to adjust for confounding in observational datasets is to calculate a propensity score.^20^ Propensity score-based methods were discussed in Part 2 of this series.^3^ In a comprehensive evaluation comparing multivariable regression with four methods of propensity scoring, multivariable regression performed as effectively as the best method of propensity scoring.^21^

## Conclusions

Regression provides a unified framework for statistical inference that has broad applications in medical research. Our goal has been to illustrate the principles of regression and to highlight the important considerations and pitfalls. For readers wishing to progress to a deeper understanding, we recommend *Regression and Other Stories* by Gelman and colleagues.^22^

## MCQs

The associated MCQs (to support CME/CPD activity) are accessible at www.bjaed.org/cme/home for subscribers to *BJA Education*.

## Declaration of generative AI and AI-assisted technologies in the writing process

During the preparation of this work, the authors used Claude (Anthropic AI) to help create the simulated datasets used in the article. After using this service, the authors reviewed and edited the content as needed and take full responsibility for the content of the published article.

## Declaration of interests

DS is an editor and editorial board member of *BJA Education*. PMJ declares no conflicts of interest.
